# Hyperglycemia-Induced Abnormalities in Rat and Human Corneas: The Potential of Second Harmonic Generation Microscopy

**DOI:** 10.1371/journal.pone.0048388

**Published:** 2012-11-05

**Authors:** Gaël Latour, Laura Kowalczuk, Michèle Savoldelli, Jean-Louis Bourges, Karsten Plamann, Francine Behar-Cohen, Marie-Claire Schanne-Klein

**Affiliations:** 1 Laboratory for Optics and Biosciences, École Polytechnique, CNRS, INSERM U696, Palaiseau, France; 2 Laboratory of Applied Optics, ENSTA ParisTech, École Polytechnique, CNRS, Palaiseau, France; 3 Team17: Physiopathology of Ocular Diseases, Therapeutic Innovations, INSERM UMRS 872, Paris, France; 4 Centre de Recherche des Cordeliers, Pierre et Marie Curie University, Paris, France; 5 Department of Ophthalmology, AP-HP Hôtel-Dieu, Paris Descartes University, Faculty of Medicine, Sorbonne Paris Cité, Paris, France; Tufts University, United States of America

## Abstract

**Background:**

Second Harmonic Generation (SHG) microscopy recently appeared as an efficient optical imaging technique to probe unstained collagen-rich tissues like cornea. Moreover, corneal remodeling occurs in many diseases and precise characterization requires overcoming the limitations of conventional techniques. In this work, we focus on diabetes, which affects hundreds of million people worldwide and most often leads to diabetic retinopathy, with no early diagnostic tool. This study then aims to establish the potential of SHG microscopy for *in situ* detection and characterization of hyperglycemia-induced abnormalities in the Descemet’s membrane, in the posterior cornea.

**Methodology/Principal Findings:**

We studied corneas from age-matched control and Goto-Kakizaki rats, a spontaneous model of type 2 diabetes, and corneas from human donors with type 2 diabetes and without any diabetes. SHG imaging was compared to confocal microscopy, to histology characterization using conventional staining and transmitted light microscopy and to transmission electron microscopy. SHG imaging revealed collagen deposits in the Descemet’s membrane of unstained corneas in a unique way compared to these gold standard techniques in ophthalmology. It provided background-free images of the three-dimensional interwoven distribution of the collagen deposits, with improved contrast compared to confocal microscopy. It also provided structural capability in intact corneas because of its high specificity to fibrillar collagen, with substantially larger field of view than transmission electron microscopy. Moreover, *in vivo* SHG imaging was demonstrated in Goto-Kakizaki rats.

**Conclusions/Significance:**

Our study shows unambiguously the high potential of SHG microscopy for three-dimensional characterization of structural abnormalities in unstained corneas. Furthermore, our demonstration of *in vivo* SHG imaging opens the way to long-term dynamical studies. This method should be easily generalized to other structural remodeling of the cornea and SHG microscopy should prove to be invaluable for *in vivo* corneal pathological studies.

## Introduction

Multiphoton microscopy is a well-established optical technique in biology because of its capability for three-dimensional (3D) multimodal imaging [1], [2]. This imaging technique is based on the non-linear interaction of a laser beam with the tissue, providing micrometer-sized optical sectioning and therefore intrinsic 3D resolution at micrometer scale. A key advantage of multiphoton microscopy is to enable the combination of different modes of contrast based on intrinsic signals in unstained tissues. In addition to two-photon excited fluorescence (2PEF) that stems from endogenous chromophores mainly located in the cytoplasm [3], second harmonic generation (SHG) can be detected from non-centrosymmetric macromolecular structures. SHG has been shown to be specific for fibrillar collagen, while non-fibrillar collagen, as type IV collagen in the basal membranes, is SHG silent [1], [2], [4], [5]. SHG microscopy therefore provides contrasted and specific images of the corneal stroma that is mainly composed of type I collagen fibrils organized in stacked lamellae and appears as a well-suited technique for cornea imaging [6]–[9]. However, this technique has been rarely used for the study of corneal pathologies, except the keratoconus [10], [11]. The main reason is the difficulty to perform *in vivo* imaging with this laser-scanning technique. *In vivo* multiphoton imaging of cornea has been reported only recently and was based on 2PEF signals from exogenous chromophores [12] and on intrinsic SHG signals using an original polarization-resolved approach to access quantitative information about the multi-scale stromal organization [13].

Diabetes mellitus is a metabolic disease characterized by chronic hyperglycemia, which affects hundreds of million people worldwide. Among the most common ocular complications, diabetic retinopathy affects a majority of diabetic people. This pathology is characterized by blood vessel formation in the retina and can lead to reduced vision and eventually to blindness. Today, there is no early diagnosis of these long-term complications. Considering that the glucose concentration in aqueous humor is correlated with the blood sugar level, various hyperglycemia-induced abnormalities have been reported in the cornea [14]–[18]. The Descemet’s membrane (DM) is located in the posterior part of the cornea between the endothelium, which is in contact with aqueous humor, and the stroma. This membrane is stratified with an anterior banded zone and a posterior unbanded zone that thickens during life. Hyperglycemia-induced DM abnormalities have thus already been reported both in diabetic rat models [19], [20] and in humans [17]. However, all these studies were based on histology and transmission electron microscopy (TEM), so that they were limited to two-dimensional (2D) *ex vivo* information after fixation and preparation of the corneal tissue.

The purpose of this study was to establish the potential of SHG microscopy for *in situ* detection and characterization of hyperglycemia-induced abnormalities in the DM. To that end, SHG microscopy was compared to confocal microscopy (CM), the gold standard in ophthalmology for *in vivo* corneal imaging at the micrometer scale [21], [22]. SHG imaging was also compared to histology to observe corneal structures in stained sections and to TEM that is the gold standard for tissue ultra-structural characterization. Similar structural corneal abnormalities were highlighted by all these imaging techniques (CM, SHG microscopy and TEM) in diabetic rat corneas and in human corneas from diabetic donors. TEM images revealed the presence of long-spacing fibrillar collagen induced by hyperglycemia, in agreement with previous reports. Most importantly, our multimodal approach showed that SHG microscopy provides 3D images of unstained corneal tissue with improved contrast and specificity compared to the other imaging techniques. Finally, *in vivo* SHG imaging of these abnormalities was demonstrated in rat. It showed the relevance of this technique for *in vivo* diagnosis of hyperglycemia-induced tissue remodeling.

## Results

**Figure 1:**
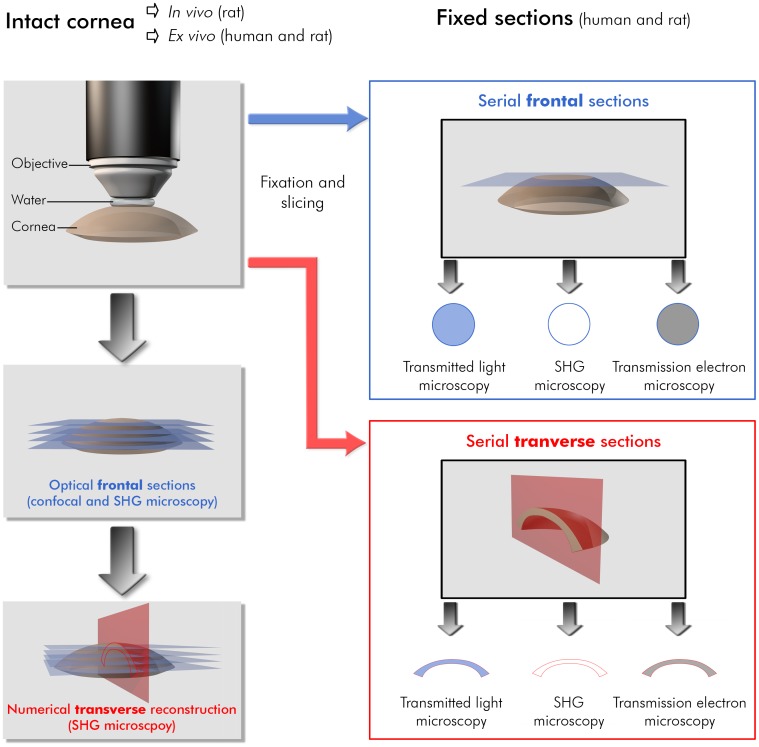
Methodology to compare the different imaging techniques on rat and human corneas. Cornea preparation, orientation of the different histological and numerical sections and imaging geometry are indicated for each imaging technique. Histological sections are unstained for SHG microscopy, stained with toluidine blue for transmitted light microscopy and stained with uranyl and lead citrate solutions for transmission electron microscopy.

In order to characterize the hyperglycemia-induced abnormalities that appear in the Descemet’s membrane (DM), we imaged rat and human corneas by use of complementary techniques. Special attention was paid to design a rigorous methodology to compare the images acquired by the different techniques. Each of these techniques is based on a specific contrast mechanism and provides images at various scales and resolutions. The protocol described in the methods insures that the same areas were imaged with all the techniques. The different techniques used, the sample preparation and the orientation of the views and of the sections are summarized in Fig. 1.

### Rat Corneas

**Figure 2:**
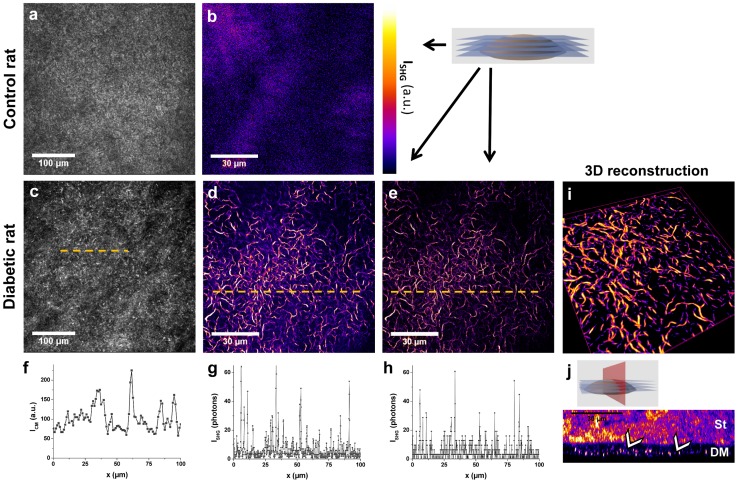
Confocal microscopy (CM) and SHG microscopy imaging of the DM from control and diabetic rat corneas. Unstained intact rat cornea observed by (a and c) *in vivo* CM and (b, d–i, j) *ex vivo* SHG microscopy detected in the (e) backward direction and in the (b,d,i–j) forward direction : (b-e) frontal optical sections, (i) 3D view and (j) transverse numerical reconstruction of the DM (image size: 108×108×7 µm^3^). Intensity profiles from (f) CM, (g) forward-detected and (h) backward-detected SHG microscopy along 100 µm are plotted under the corresponding images. The number of detected photons has been corrected from the channel sensitivity. St: stroma, DM: Descemet’s membrane. White arrows indicate collagen abnormal deposits in the DM. The look-up-table (LUT) used for SHG images is indicated near (b).

Figure 2 compares images obtained by *in vivo* confocal microscopy (CM) and *ex vivo* second harmonic generation (SHG) microscopy, from the same corneas of a control rat and a diabetic rat (see Table 1). It corresponds to *in situ* observations of the entire cornea. No signal was detected in the DM from control rats using CM (Fig. 2a) in agreement with previous reports [21]. In contrast, *in vivo* CM revealed poorly defined hyper-reflective micrometric structures in the posterior cornea from diabetic animals (Fig. 2c). Axial scanning (along the depth) performed during *in vivo* imaging showed that these structures were located within the DM because they appeared below the stroma, which is readily located using the reflection of keratocyte nuclei [21], [22], and above the endothelium characterized by the endothelial cells mosaic. *Ex vivo* SHG images exhibited strong signals in the same region of the intact excised unfixed cornea both in forward (Fig. 2d and Video S1) and backward (Fig. 2e) detection directions. SHG images have much better contrast than CM as shown in the intensity profiles in Fig. 2f–h. This region was readily located below the stroma that is characterized by striated SHG images and above the endothelium that shows low 2PEF signal from endothelium cells [9]. In contrast, the DM from control animals showed no SHG signal as expected for a non fibrillar matrix (Fig. 2b). By recording image stacks along the corneal depth, the spatial extent of these abnormal structures was evaluated to around 5 µm, as shown in transverse reconstruction (Fig. 2 h and Video S2) and 3D view (Fig. 2g and Video S3). The transverse reconstruction exhibited these highly contrasted structures on a SHG-silent background in the DM. Note that the strong signals in the upper part of the image correspond to the stromal collagen.

**Table 1:** Diabetic and control rat corneas: clinical data and observations by SHG microscopy and other imaging techniques.

Sex	Animal	Blood glucose level (mg/mL)	Age (months)	Cornea	Presence of collagen(SHG microscopy)		Other imaging techniques
					*center*	*periphery*	
**Goto-Kakizaki rats**							
M	1	507	12	1a right	−	+++	CM
				1b left	−	+++	CM, histology, TEM
	2	559	14	2a left	−	+++	
F	3	231	12	3a right	−	+++	CM
				3b left	−	+++	CM
	4	220	12	4a left	+	+++	CM, histology, TEM
	5	422	14	5b right	−	+++	Histology, TEM
**Wistar rats**							
M	6	N/A	5	6a left	−	−	CM
	7	N/A	14	7a left	+	+	CM, histology
	8	N/A	5	8a right	−	−	CM
		N/A		8b left	−	−	CM

Grading is as follows: N/A: not available, −: absence of fibrillar collagen, +: few fibrillar collagen, ++: great concentration of fibrillar collagen, +++: fibrillar collagen over the whole field of view.

**Figure 3:**
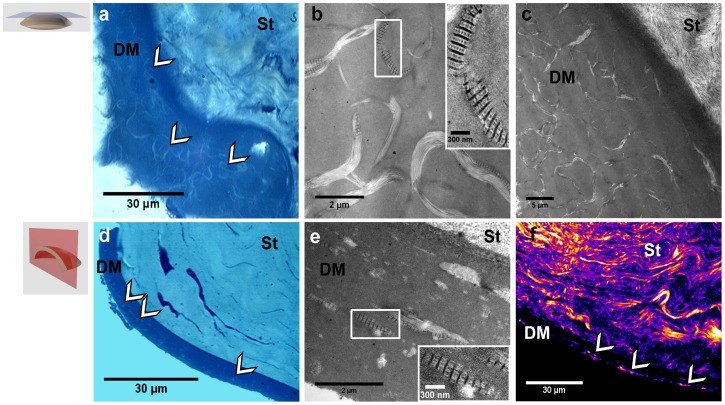
Multimodal imaging of histological sections from the same diabetic rat cornea. (a–c) Frontal and (d–f) transverse histological sections of the cornea observed (a, d) by transmitted light microcopy, (b, c and e) by TEM, where insets show long-spacing collagen, and (f) by SHG microscopy. St: stroma, DM: Descemet’s membrane. White arrows indicate collagen abnormal deposits in the DM.

After tissue fixation, serial transverse and frontal histological sections were prepared to perform complementary observations (Fig. 3a–c and d–f respectively). Stained sections observed by transmitted-light microscopy revealed the above abnormal structures, but with low contrast. On the frontal view (Fig. 3a), the spatial interwoven organization of the abnormalities could be easily recognized and the transverse view was similar to the transverse reconstruction of the SHG images (Fig. 3d). Unstained thin sections were then visualized by SHG microscopy (Fig. 3f). The stroma was identified in the upper part by its lamellar collagen organization with strong SHG signals. Discontinuous SHG signals were also observed in the DM (see arrows) and formed similar images as the transverse reconstruction obtained by *in situ* observation of the cornea (Fig. 2 h). Because of the increased lateral resolution compared to the axial one, posterior stromal organization was better visualized in this transverse section than in the previous reconstruction. TEM observations of the same area from the following serial sections revealed striated collagen fibrils along both orientations (Fig. 3b, c and e). It is worth noting that the period of the banding pattern was 120 nm instead of 67 nm for usual collagen fibrils (see inset Fig. 3b and e). It showed that the deposits were composed of long-spacing collagen [20], [23].

The above observations were confirmed by imaging the other corneas from GK rats (n = 5). All diabetic rats exhibited collagenous deposits in the DM characterized by strong SHG signals and interwoven spatial organization. They were only observed in the periphery of the corneas and not in the center (see Table 1 for a complete description of the observations performed). Corneas from non-diabetic rats (n = 3) did not exhibit these deposits, except for a few oldest animal where sparse deposits were also observed.

### Human Corneas

**Figure 4:**
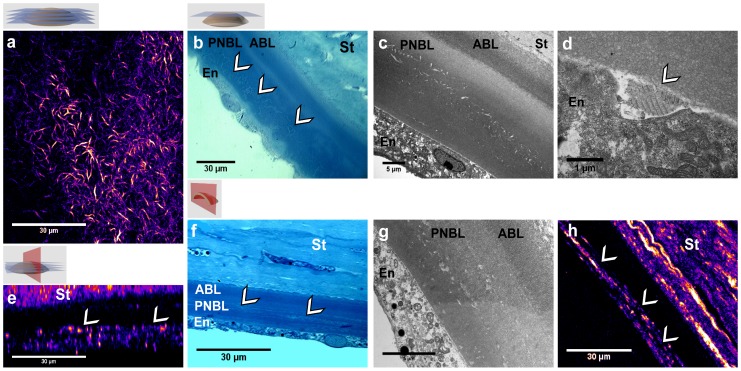
Multimodal imaging of the DM from the same diabetic human cornea. (a–d) Frontal and (e–h) transverse sections. (a, e) SHG microscopy of intact cornea: (a) frontal optical section, (e) transverse numerical reconstruction. (b, f) Transmitted light microcopy of stained histological sections, where the abnormalities are visible with few contrast. (c, d, g) TEM views of (c, g) the entire DM and (d) its posterior part and the endothelium: long-spacing collagen appears to be synthesized by the endothelial cell. (h) SHG imaging of the same transverse histological section, where fibrillar collagen is clearly identified in the DM. St: stroma, ABL: anterior banded layer, PNBL: posterior nonbanded layer, En: endothelium. White arrows indicate collagen abnormal deposits in the DM.

Corneas from diabetic human donors were studied using the same multimodal imaging protocol. To illustrate the interest of the various imaging techniques, Figure 4 displays typical images of a cornea obtained from a 83 year-old donor with unbalanced type 2 diabetes (see cornea No. 2 in Table 2).

**Table 2:** Human corneas: clinical data and observations by SHG microscopy and other imaging techniques.

Cornea	Age (years)/sex	Endothelial cell density (cells/mm^2^)	Clinical data	Presence of collagen(SHG microscopy)		Other imaging techniques
				*center*	*periphery*	
**Diabetes**						
1	80/M	2450	unbalanced type 2 diabetes	++	N/A	TEM
2	83/M	1850	unbalanced type 2 diabetes	+++	+++	Histology, TEM
3a	83/F	1900	balanced type 2 diabetes	−	+	
3b		1650		+	+	
**Hypertension**						
4a	78/F	1600	hypertension	N/A	+++	Histology, TEM
4b		1900		N/A	++	Histology, TEM
5	65/M	2600	hypertension	N/A	N/A	TEM
6	88/M	1850	hypertension	N/A	N/A	Histology, TEM
**Control**						
7	60/F	2450		−	−	
8	65/M	1800		−	+	

Grading is as follows: N/A: not available, −: absence of fibrillar collagen, +: few fibrillar collagen, ++: great concentration of fibrillar collagen, +++: fibrillar collagen over the whole field of view.

*In situ* SHG imaging was first performed in intact unfixed corneas. Strong SHG signals were detected in the DM and showed an interwoven spatial organization (Fig. 4a and Video S4). Transverse reconstruction revealed that these abnormal structures extended along several micrometers through the DM and were located 5 to 10 µm beneath the posterior stroma (Fig. 4e). SHG microscopy allowed 3D reconstruction of these structures as shown in Video S5. These structures were observed at any given position of the cornea, both in the periphery and in the center. It is worth noting that the spatial organization and the typical size of these deposits were similar to the ones observed in the rat corneas.

Secondly, the tissue was fixed and sliced along transverse and frontal sections for further insight into these deposits. Unstained thin sections exhibited strong SHG signals and confirmed the location of the abnormalities within the DM (Fig. 4h), while the deposits were observed with low contrast in histology (Fig. 4b and f). TEM imaging of ultra-thin sections highlighted fibrillar collagenous structures in the postnatal DM or posterior nonbanded layer (PNBL) (Fig. 4c and g). Note that postnatal DM is located above the endothelium and grows over time to reach 10 to 20 µm thickness, while prenatal DM, or anterior banded layer (ABL), corresponds to the first micrometers just beneath the posterior stroma. TEM images at a larger magnification (Fig. 4d) revealed long-spacing collagen with 120 nm wide banding pattern in some parts of the deposits. Most interestingly, these structures were observed in the very proximity of the endothelium, suggesting that they were synthesized by the endothelial cells (arrow in Fig. 4d).

**Figure 5:**
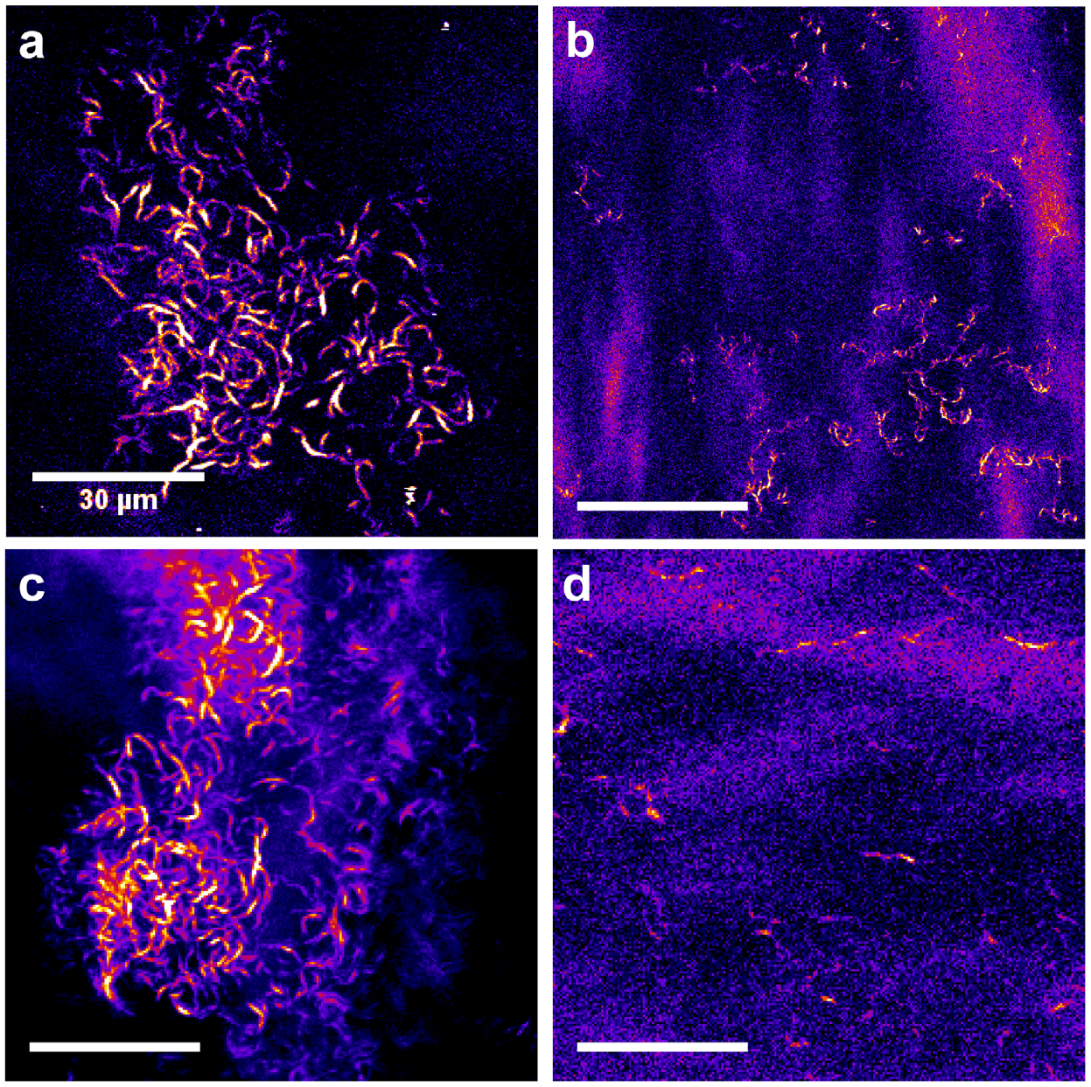
SHG imaging of the DM from human corneas with different clinical data. Transverse optical section in the DM (a) from a diabetic donor with unbalanced type 2 diabetes, (b) from a diabetic donor with balanced type 2 diabetes, (c) from a donor with hypertension and (d) from a donor without clinical data related to hyperglycemia or hypertension.

Finally, corneas from donors with various clinical data, including unbalanced and balanced type 2 diabetes and hypertension, have been compared (see Fig. 5). When the diabetes was balanced, the density of the fibrillar collagen deposits in the DM observed by SHG was quite low (Fig. 5b). On the contrary, SHG imaging of corneas from hypertensive donors also exhibited collagen deposits in the DM (Fig. 5c), with the same spatial organization as for unbalanced diabetic donors (Fig. 5a). Finally, corneas from donors without hypertension or diabetes according to their clinical data did not exhibit any SHG signal in the center of the DM and only few deposits in the periphery of the tissue (Fig. 5d).

These results are summarized in Table 2. They show that diabetes and hypertension induce the formation of abnormal collagen deposits in the DM with a interwoven spatial organization, as readily observed with SHG microscopy, while in control corneas, DM do not exhibit any SHG signal.

### *In vivo* SHG Imaging of Rat Corneas

**Figure 6:**
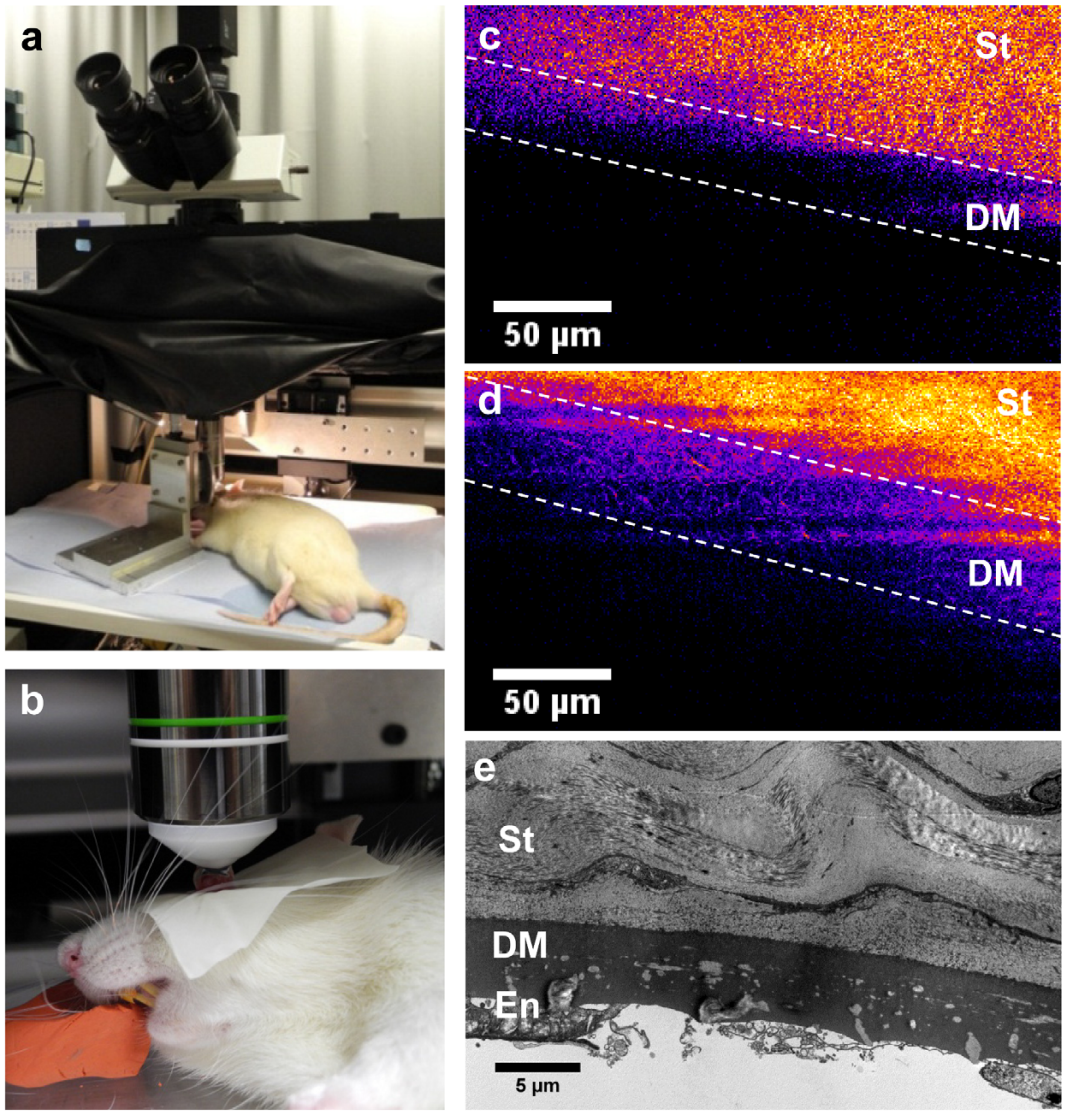
*In vivo* SHG imaging of the DM from a rat cornea. (a–b) experimental setup for *in vivo* imaging of the anesthetized rat (backward detection). (c–d) *In vivo* SHG observation of DM (between the dashed lines) (c) without any SHG signal in the control rat and (d) with SHG signals from fibrillar collagen in the diabetic rat. (e) TEM observation of DM collagen deposits after sacrifice of the diabetic rat.

Finally, our multiphoton microscope was adjusted to perform *in vivo* corneal imaging (Fig. 6a and b). The trans-detection channel was removed and the pixel acquisition rate was increased to 300 kHz that is a few tens of a second per frame. *In vivo* stromal imaging was characterized by strong homogenous SHG signals [13]. To focus on the DM, imaging was performed along the whole depth of the stroma to precisely locate the posterior stroma. Whereas no SHG signals were detected in the control rat (Fig. 6c), abnormal structures in the DM were visualized in GK rat across the cornea despite the vital movements of the animal (Fig. 6d). *In vivo* backward-detected SHG signals were weaker than the previous ones that were recorded *ex vivo* in a forward-detected channel. Nevertheless, the interwoven abnormal structures were still unambiguously identified.

This observation was confirmed by *ex vivo* SHG imaging of the same cornea mounted between two coverslips after the sacrifice of the animal (data not shown), which showed a dense distribution of deposits in the periphery of the cornea as observed in other animals (see Fig. 3). Finally, TEM of ultra-thin sections also revealed the high concentration of these deposits (Fig. 6e) and confirmed that some of these structures were long-spacing collagen with 120 nm-wide banded pattern.

## Discussion

### Comparison of the Different Imaging Techniques: the Potential of SHG Microscopy

Hyperglycemia-induced abnormalities in the DM were observed with different imaging techniques to point out the advantages and drawbacks of each of these techniques and to determine their potential for diagnosis.

CM is routinely used in ophthalmology nowadays, mainly to evaluate the endothelial cell density. This optical technique provides 3D images of unstained tissues by the use of a pinhole in front of the detector to achieve optical sectioning. The contrast of confocal images stems from the spatial variation of the refractive index. In addition to endothelium observation, CM is also used for observation and quantification of the epithelial cells, nerve plexus, and keratocytes [21], [22]. We showed in this study that *in vivo* CM can be used to evidence abnormalities in the DM of rat corneas. However, endothelial cells that are adjacent to the DM gave rise to reflection signals with a similar order of magnitude. This explains why the intensity profile in Fig. 2e exhibits strong background signal. Consequently, CM provided poorly contrasted images of these abnormalities and lacked specificity. Note that Optical Coherence Tomography (OCT), that is another well-established optical technique in ophthalmology [24], exhibits the same drawbacks. While optical sectioning is performed in a different way as for CM, by use of interferometry, this imaging technique is based on the same physical contrast as CM and the observed structures are intrinsically similar [25], [26]. Thus, neither better contrast nor better specificity is expected by using OCT instead of CM.

On the contrary, multiphoton microscopy provides multimodal cell-scale imaging of unstained tissues with high specificity. 2PEF signals are specific for cells (epithelial cells, keratocytes and endothelial cells), SHG for fibrillar collagen [13] and Third Harmonic Generation (THG) for cell-cell junctions, nuclear membranes and stromal intralamellar interfaces [9]. Regarding the hyperglycemia-induced abnormalities, our study shows that the deposits in the DM exhibit strong SHG signal. The DM is mainly composed of type- IV and -VIII collagen [27] that form networks and are SHG silent [4]. This was confirmed by observations in rat and human control corneas that exhibited no SHG signal in the DM. In contrast, *ex vivo* SHG microscopy of diabetic corneas provided well contrasted and background-free images of the collagen deposits in the DM as shown on the intensity profile in Fig. 2f. This is the first *in situ* characterization of these hyperglycemia-induced abnormalities. SHG images show similar patterns in forward and backward directions as expected from isolated fibrils. Note that SHG microscopy can detect single collagen fibrils with diameters above 50–100 nm typically [28]. It means that *in situ* observation by use of this optical technique is sensitive to collagen deposits with diameter below the optical resolution. Previous studies already underlined the formation of these collagenous structures by use of TEM [17], [20], [29]. However, TEM images were limited to 2D sections with a small field of view and could suggest that these structures were linear. In contrast, 3D reconstruction of SHG images allowed the spatial characterization of these structures and showed for the first time that the collagen fibrillar deposits were interwoven in a meshlike arrangement.

TEM is the gold standard imaging technique of the ocular ultra-structure. The main drawback of this technique is that it requires a time-consuming sample preparation and provides only a small field of view of the tissue. Nevertheless, this technique is crucial to characterize and to decipher biological processes with a better insight at the nanometer scale. In this regard, our multimodal approach combining optical and electron microscopies appears as highly efficient. While SHG provides *in situ* 3D information about the whole cornea as discussed above, TEM enables to focus on areas of interest and to confirm the precise location of the deposits in the postnatal DM, to determine the ultra-structure of these collagen deposits that appeared as long-spacing collagen (Fig. 3b and e), and to get insight into biological processes, such as the production of these deposits by endothelial cells (Fig. 4d).

Until recently, SHG studies of the cornea have been mainly performed *ex vivo*. *In vivo* 2PEF imaging has been recently reported in rat corneas but used exogenous labels [12], [30]. Most interestingly, we demonstrated recently the feasibility of *in vivo* imaging of the stromal lamellae architecture in unstained corneas [13]. In the present study, we demonstrated *in vivo* SHG imaging of pathological corneas. We successfully detected the formation of collagen deposits in the DM despite the vital movements of the rat. The snapped images were slightly blurred and the setup still needs some technical improvements to enable routine detection of these abnormalities and quantitative *in vivo* analyses. In particular, an immobilization device could be implemented in a straightforward way like in *in vivo* CM. Specific lenses, such as Gradient-index lenses, could also be used to better access the corneal periphery. Altogether, SHG microscopy appears as a reliable technique for *in vivo* observations because it is based on background-free endogenous signals and is therefore quite sensitive. This first demonstration of *in vivo* SHG imaging of DM abnormalities thus opens avenues for long-term dynamical studies.

### Hyperglycemia-induced Abnormalities in the DM

Our study shows that DM from control animals and human donors without any reported hyperglycemia were free of fibrillar collagen. A few studies reported the formation of long-spacing fibrillar collagen within the DM in the case of iridocorneal-endothelial syndrome [23], [29] and of Fuch’s dystrophy [23], [31]. Previous studies in GK rats showed that these long-spacing fibrils are composed of type VIII collagen [20], [23]. These studies pointed out several concomitant factors such as the accumulation of advanced glycation end products (AGEs) [32] and oxidative stress [33]. Our data confirmed these observations and provided complementary information about the interwoven spatial arrangement of these long-spacing collagen fibrils and their extent within the DM. In particular, SHG mapping of the whole DM showed that these abnormalities are formed mainly in the periphery of the DM. Most importantly, fibrillar collagen abnormalities appear to be produced by the endothelial cells as suggested in Fig. 4d. Accumulation of these abnormalities in the posterior part of the DM, which corresponds to the PNBL, is also clearly observed in TEM images of human corneas from diabetic donors. The high glucose level in the aqueous humor in case of chronic hyperglycemia could indeed induce intracellular hyperglycemia in endothelial cells and subsequent extracellular matrix modification as suggested in [34]. Characterization of the spatial extent of these abnormal deposits by SHG microscopy should then enable the quantitative assessment of antidiabetic agents that could limit the formation of these abnormalities [20].

Our study demonstrates that these abnormal deposits can be detected by *in vivo* multiphoton microscopy, which should enable long-term dynamical studies in animal models. Such studies should give complementary information about the dynamic of the involved processes. To that end, type 1 diabetes could be induced by streptozotocin injection in Wistar rats and the formation of the abnormal fibrils in the DM could be monitored by *in vivo* multiphoton microscopy and correlated to physiological parameters. The key point of such a study would be to establish a chronology of the development of abnormalities in the cornea and in the retina in order to determine whether SHG imaging of corneal abnormalities could serve as an early diagnostic tool of diabetic retinopathy. It would then further support the use of SHG microscopy for *in vivo* detection of pathologic collagen in the DM of unstained corneas.

### Conclusion

Our study demonstrates unambiguously the high potential of SHG microscopy for 3D characterization of corneal structural abnormalities. This optical imaging technique requires no specific tissue preparation and provides 3D capability and *in vivo* feasibility like CM. Nevertheless, SHG images exhibit much enhanced contrast due to the high specificity of SHG process for fibrillar collagen, while cells and nonfibrillar matrix are SHG silent. Hyperglycemia-induced collagen deposits in the DM then appear as background-free contrasted structures, while CM images show background signals from a variety of other structures. Moreover, the high structural specificity of SHG microscopy provides complementary information about the sub-micrometer fibrillar organization of these collagen deposits, as confirmed by TEM with a smaller field of view. Finally, SHG imaging of rat and human corneas reveals in a unique way the 3D interwoven distribution of these collagen deposits and shows that they are located in the posterior part of the DM periphery. This method should be easily generalized to other structural remodeling of the cornea and SHG microscopy should prove to be invaluable for *in vivo* corneal pathological studies.

## Materials and Methods

### Rats

Experiments were conducted in accordance with the Association for Research in Vision and Ophthalmology (ARVO) Statement for the Use of Animals in Ophthalmic and Vision Research. The study was approved by the Regional Ethics Committee in Animal Experiment N°3 of Ile-de-France region (approval p3/2008/062). Five (12 to 14-month old) Goto-Kakizaki (GK) rats, a spontaneous model of type 2 diabetes from our facility, and three age-matched (5 and 14-month old) non-diabetic Wistar animals, purchased from Janvier (Le Genest-Saint-Isle, France), were included in this study. Information related to the animals and to the performed experiments is summarized in Table 1. Animals were roomed for one week before inclusion in the study. They were anesthetized by intramuscular injection of a mixture of Ketamine (100 mg/kg) and Xylazine (10 mg/kg) for *in vivo* experiments (confocal microscopy (CM) and multiphoton microscopy). After the instillation of a local anesthetic (tetracaine) in the cornea, the optical contact was maintained with an ophthalmic gel (Lacrigel, Europhta, Monaco).

### Human Cornea Preparation

The study was conducted according to the tenets of the Declaration of Helsinki and French legislation for scientific use of human corneas. Humans corneas were obtained from the French Eye Bank (BFY, Paris, France), which were unsuitable for transplantation and assigned to scientific use [35]. 10 corneas from 8 donors, 4 males and 4 females, 66 to 88 year old, were included in this study. Age, sex and clinical data of the donors are summarized in Table 2. Corneas were stored at 31°C in CorneaMax medium (Eurobio, France) until imaging experiments.

### *In vivo* Confocal Microscopy

*In vivo* CM was performed with the Heidelberg Retina Tomograph II using the Rostock Cornea Module (Heidelberg Engineering GmbH, Heidelberg, Germany) with a sterile cap and with ophthalmic gel between the microscope and the cornea. Image acquisition time was 24 ms, providing video rate imaging. The field of view was 400×400 µm^2^ for 384×384 pixels, that is 1.05 µm/pixel. *In vivo* CM was realized in the center and then in the periphery in four characteristic areas (nasal, temporal, superior and inferior).

### Multiphoton Microscopy

A custom-built laser-scanning upright multiphoton microscope was used for second harmonic generation (SHG) imaging [9], [13], [36]. The light source was a femtosecond titanium-sapphire laser (Tsunami, Spectra-Physics) tuned at 860 nm. High numerical aperture water immersion lenses (20×, NA 0.95 and 60×, NA 1.2, Olympus) were used to achieve 0.4 µm lateral- × 1.6 µm axial-resolution and 0.3 µm lateral-×0.9 µm axial-resolution respectively near the tissue surface. SHG signals, which are emitted at twice the excitation frequency that is half the excitation wavelength, were selected using spectral filters (FF01-427/10 interferential filter and 680SP short-pass filters, Semrock) and detected using photon-counting photomultiplier tubes (P25PC, Electron Tubes). SHG was detected in the backward and forward directions for *ex vivo* cornea (rat and human) imaging and only in the backward direction for *in vivo* imaging (rat). Unless otherwise specified (in Fig. 2 and Fig. 6), all figures corresponds to forward-detected SHG images. The field of view was typically 110×110 µm^2^ with 0.8 to 0.25 µm pixel size. The pixel acquisition time was a few µs.

Rat corneas were removed just after the sacrifice of the animal and were mounted between two glass coverslips for *ex vivo* imaging (no fixation). The same areas as the previous ones observed by *in vivo* CM were observed by SHG microscopy. Human corneas were mounted unfixed in the same way. Laser power for *ex vivo* observations was 30 to 50 mW under the objective. SHG observations were then performed at the center and in the periphery of the tissue. For *in vivo* imaging, ophthalmic gel was used to maintain optical contact between the objective and the cornea. The laser power was 115 mW in this case.

### Histology and Transmission Electron Microscopy

After multiphoton imaging, corneas were fixed for 2 hours in 2.5% glutaraldehyde in 0.1 M cacodylate buffer (pH 7.4), then in 1% osmium tetraoxide in 0.2 M cacodylate buffer (pH 7.4) and dehydrated in successive graduated ethanol solutions and in propylene oxide. Areas of interest were included in epoxy resin. To optimize the comparison between the various imaging techniques, these blocks were oriented along transverse sections (as classically used in histology) and frontal ones (as observed in CM and SHG microscopy) as described in Fig. 1. Serial sections were then obtained from every block with an ultramicrotome (OmU2, Reichert, Austria) and used to compare images from histology, multiphoton microscopy and TEM and to insure the colocalization of the structures of interest. First, semi-thin sections (1 to 2 µm thick) were stained with toluidine blue and observed with conventional transmitted light optical microscopy, while other ones were mounted unstained for multiphoton imaging. Second, ultrathin sections (around 100 nm thick) were stained with uranyl and lead citrate solutions and examined with TEM (CM10, Philips, The Netherlands) coupled to a CCD camera (ES1000W Erlangshen, Gatan, France).

## Supporting Information

Video S1 *Ex vivo* SHG microscopy from the posterior cornea of a diabetic rat. Frontal optical sections are recorded at increasing depth from the posterior stroma (striations all over the field of view) to the Descemet’s membrane (micrometer-sized deposits). Scale bar: 30 µm, field of view: 108×108 µm^2^, z-step: 0.5 µm.
(AVI)

Video S2 *Ex vivo* SHG microscopy from the posterior cornea of a diabetic rat: transverse numerical reconstructions. Scale bar: 30 µm, field of view: 108×30 µm^2^, y-step: 0.25 µm.
(AVI)

Video S3 *Ex vivo* SHG microscopy from the Descemet’s membrane of a diabetic rat, 3D views: 108×108×7 µm^3^.
(AVI)

Video S4 *Ex vivo* SHG microscopy from the posterior cornea of a diabetic human donor. Frontal optical sections are recorded at increasing depth from the posterior stroma (striations all over the field of view) to the Descemet’s membrane (micrometer-sized deposits). Scale bar: 30 µm, field of view: 90×90 µm^2^, z-step: 0.5 µm.
(AVI)

Video S5 *Ex vivo* SHG microscopy from the Descemet’s membrane of a diabetic human donor, 3D views: 90×90×13 µm^3^.
(AVI)
